# Structural and Biomechanical Properties of Supramolecular Nanofiber-Based Hydrogels in Biomedicine

**DOI:** 10.3390/biomedicines12010205

**Published:** 2024-01-17

**Authors:** Raffaele Pugliese

**Affiliations:** NeMO Lab, ASST GOM Niguarda Cà Granda Hospital, 20142 Milan, Italy; raffaele.pugliese@nemolab.it

The field of supramolecular nanofiber-based hydrogels in biomedicine has witnessed remarkable growth over the past two decades [1]. The versatility of these materials, characterized by their modularity, ease of modification, and ability to mimic the native extracellular matrix (ECM) of living tissues, positions them as pivotal players in various biomedical applications [2]. For these reasons, supramolecular nanofibrous hydrogels have become a leading strategy for creating functional biomaterials that are useful for different applications, including (1) three-dimensional cell cultures or organoids of primary and stem cells [3]; (2) the sustained release of drugs, small molecules, bioactive molecules, growth factors, and siRNA [4]; (3) hemostat solutions [5]; (4) fillers or injectable scaffolds for regenerative medicine, as well as tissue engineering; (5) 3D printing [6]; and (6) actuators for optics and fluidics. Furthermore, a few supramolecular hydrogels have been used in clinical trials for wound healing, treating cancers, and surgical use [7]. It is likely that such materials can open doors for different directions in the areas of tissue engineering, synthetic biology, biomedicines, and food chemistry, to name a few.

This Special Issue focuses on the latest advances in biomaterials, emphasizing improved structural, mechanical, and nano-architectural features with the potential to revolutionize biomedicine and more.

The paper by Pitz et al., explores the use of self-assembling peptides for converting Temozolomide (TMZ) and delivering a model drug into glioblastoma multiforme cells (Contribution 1). This study highlights the potential of peptide-based drug delivery systems to create local stimuli during drug delivery while maintaining biocompatibility, offering promising avenues for cancer treatment and more.

In a comparative evaluation, Hilal and colleagues investigate the structural and biomechanical properties of food-grade biopolymers (i.e., pea protein, wheat protein, gellan gum, konjac gum, inulin, maltodextrin, psyllium, and tara gum) as potential hydrogel building blocks (Contribution 2). This work provides valuable insights into the properties of different biopolymers, laying the foundation for their potential applications in hydrogel-based biomedical technologies.

In the realm of tissue engineering and regenerative medicine, contributions from Halperin-Sternfeld et al. [8] delve into the development of thixotropic red microalgae sulfated polysaccharide-fluorenylmethoxycarbonyl diphenylalanine peptide composite hydrogels as tunable scaffolds for tissue engineering that may allow cell differentiation into various lineages (Contribution 3). By combining the unique bioactivities of sulfated polysaccharides with the structural and mechanical properties of peptides, this study introduces tunable and injectable hydrogels with potential applications in controlled drug release.

Additionally, the study by Ferreira et al. explores the wound-healing potential of a gel based on chicha gum, chitosan, and *Mauritia flexuosa* oil (Contribution 4). The formulation demonstrates antimicrobial, antioxidant, and anti-inflammatory activities, providing a new approach to wound healing with the support needed for a natural healing process.

Regarding the bioengineering of nanonutraceuticals, Pugliese and colleagues present an innovative approach by functionalizing self-assembling peptides with food-bioactive motifs (Contribution 5). This work explores the nanostructures, biomechanics, and biological features of these peptides, showcasing their potential as functional biomaterials with DPP-IV and ACE inhibitory activity, thereby contributing to the prevention of metabolic syndrome.

Furthermore, the nanostructure, self-assembly, mechanical properties, and antioxidant activity of a lupin-derived peptide hydrogel are explored by the same authors. This study addresses the challenges associated with naturally occurring food peptide-based hydrogels, offering a new tool to fine-tune mechanical properties and tailor antioxidant activities with implications for food chemistry, biochemistry, and bioengineering [9] (Contribution 6).

Lastly, regarding clinical applications of nanofibers-based hydrogels, the review by Bayer [10] provides a comprehensive overview of sustained drug release studies from nanofiber hydrogels, emphasizing the potential of these materials in biomedical applications (Contribution 7). The integration of nanofibers with hydrogels, especially in drug delivery, opens new avenues for prolonged release, with implications for cancer treatment and other therapeutic applications.

Instead, the comprehensive review by Chiew Ng et al. focuses on the potential of tissue engineering as a promising treatment for glottic insufficiency (Contribution 8). This article discusses biomolecules and cell-laden hydrogels injected into the biological system, highlighting the cost-effectiveness and protective role of injectable biomaterials.

In conclusion, this Special Issue presents a comprehensive collection of original works and reviews, showcasing the breadth and depth of research in the field of supramolecular nanofiber-based hydrogels in biomedicine. The diverse applications discussed, ranging from drug delivery and tissue engineering to wound healing and functional biomaterials, underscore the multifaceted potential of these innovative materials. As we continue to unravel the intricacies of nanofiber-based hydrogels, we anticipate further breakthroughs that could propel the field toward new horizons in biomedicine and related disciplines.

## List of Contributions

1. Pitz, M.; Elpers, M.; Nukovic, A.; Wilde, S.; Gregory, A.J.; Alexander-Bryant, A. De Novo Self-Assembling Peptides Mediate the Conversion of Temozolomide and Delivery of a Model Drug into Glioblastoma Multiforme Cells. *Biomedicines* **2022**, *10*, 2164. https://doi.org/10.3390/biomedicines10092164.
2. Hilal, A.; Florowska, A.; Florowski, T.; Wroniak, M. A Comparative Evaluation of the Structural and Biomechanical Properties of Food-Grade Biopolymers as Potential Hydrogel Building Blocks. *Biomedicines* **2022**, *10*, 2106. https://doi.org/10.3390/biomedicines10092106.
3. Halperin-Sternfeld, M.; Netanel Liberman, G.; Kannan, R.; Netti, F.; Ma, P.X.; Arad, S.M.; Adler-Abramovich, L. Thixotropic Red Microalgae Sulfated Polysaccharide-Peptide Composite Hydrogels as Scaffolds for Tissue Engineering. *Biomedicines* **2022**, *10*, 1388. https://doi.org/10.3390/biomedicines10061388.
4. Ferreira, M.O.G.; Ribeiro, A.B.; Rizzo, M.S.; de Jesus Oliveira, A.C.; Osajima, J.A.; Estevinho, L.M.; Silva-Filho, E.C. Potential Wound Healing Effect of Gel Based on Chicha Gum, Chitosan, and *Mauritia flexuosa* Oil. *Biomedicines* **2022**, *10*, 899. https://doi.org/10.3390/biomedicines10040899.
5. Pugliese, R.; Bartolomei, M.; Bollati, C.; Boschin, G.; Arnoldi, A.; Lammi, C. Gel-Forming of Self-Assembling Peptides Functionalized with Food Bioactive Motifs Modulate DPP-IV and ACE Inhibitory Activity in Human Intestinal Caco-2 Cells. *Biomedicines* **2022**, *10*, 330. https://doi.org/10.3390/biomedicines10020330.
6. Pugliese, R.; Arnoldi, A.; Lammi, C. Nanostructure, Self-Assembly, Mechanical Properties, and Antioxidant Activity of a Lupin-Derived Peptide Hydrogel. *Biomedicines* **2021**, *9*, 294. https://doi.org/10.3390/biomedicines9030294.
7. Bayer, I.S. A Review of Sustained Drug Release Studies from Nanofiber Hydrogels. *Biomedicines* **2021**, *9*, 1612. https://doi.org/10.3390/biomedicines9111612.
8. Ng, W.C.; Lokanathan, Y.; Baki, M.M.; Fauzi, M.B.; Zainuddin, A.A.; Azman, M. Tissue Engineering as a Promising Treatment for Glottic Insufficiency: A Review on Biomolecules and Cell-Laden Hydrogel. *Biomedicines* **2022**, *10*, 3082. https://doi.org/10.3390/biomedicines10123082.
